# Genetic and Clinical Factors Associated with Olokizumab Treatment in Russian Patients with Rheumatoid Arthritis

**DOI:** 10.3390/jpm12040641

**Published:** 2022-04-15

**Authors:** Dmitry S. Mikhaylenko, Ekaterina B. Kuznetsova, Viktoria V. Musatova, Irina V. Bure, Tatiana A. Deryagina, Ekaterina A. Alekseeva, Vadim V. Tarasov, Andrey A. Zamyatnin, Marina V. Nemtsova

**Affiliations:** 1 Laboratory of Medical Genetics, I.M. Sechenov First Moscow State Medical University (Sechenov University), 119991 Moscow, Russia; dimserg@mail.ru (D.S.M.); kuznetsova.k@bk.ru (E.B.K.); bureira@mail.ru (I.V.B.); katrina_5@inbox.ru (E.A.A.); nemtsova_m_v@mail.ru (M.V.N.); 2 Laboratory of Epigenetics, Research Centre for Medical Genetics, 115522 Moscow, Russia; shkarupo@mail.ru (V.V.M.); t.deryagina2018@yandex.ru (T.A.D.); 3 Institute of Translational Medicine and Biotechnology, I.M. Sechenov First Moscow State Medical University (Sechenov University), 119991 Moscow, Russia; tarasov-v-v@mail.ru; 4 Belozersky Institute of Physico-Chemical Biology, Lomonosov Moscow State University, 119992 Moscow, Russia; 5 Department of Biotechnology, Sirius University of Science and Technology, 1 Olympic Ave, 354340 Sochi, Russia; 6 Faculty of Health and Medical Sciences, University of Surrey, Guildford GU2 7X, UK

**Keywords:** rheumatoid arthritis, olokizumab, genetic predisposition, genotyping, NGS, response to therapy

## Abstract

Rheumatoid arthritis (RA) is a chronic systemic inflammatory disease and its treatment is an urgent problem of rheumatology. Olokizumab (OKZ) is a new humanized monoclonal antibody targeting IL-6 and is one of the few promising drugs for RA therapy. One-hundred-and-twenty-five DNA samples from Russian patients with RA, treated with olokizumab, were genotyped with an NGS panel containing 60 single nucleotide polymorphisms (SNPs) and the whole coding sequences of *IL6*, *IL6R*, *TNFRSF1A*, *CTLA4*, *IL10*, *IL23R*, and *PADI4*; and by RT-PCR for HLA-DRB1 and HLA-B. Associations of polymorphic variants with olokizumab efficacy according to the scores ACR20, ACR50, and DAS28-CRP were determined. We analyzed the obtained data by using logistic regression, ROC curves, and multivariate ANOVA. A high predictive value of the response to olokizumab therapy at 24 weeks was found for the combination of HLA-DRB1*04 and HLA-B*27 alleles with SNPs located in non-HLA genes (*IL1B*, *IL17A*, *PADI4*, *DHODH*, *GLCCI1*, *IL23R*, and *TNFAIP3*), and clinical characteristics (age, RA duration, and intensity) according to ACR20. Thus, the comprehensive assessment of polymorphic variants of HLA and non-HLA genes considering population characteristics in combination with clinical parameters allows for the elaboration of an RA prognostic panel.

## 1. Introduction

Rheumatoid arthritis (RA) is a chronic systemic inflammatory disease of the connective tissue with progressive joint damage and autoimmune disorders. RA is considered as a multifactorial disease associated with genetic predisposition and the effect of adverse factors (smoking, particulate matter air pollution, and organic pollutants with bacterial endotoxins) [1]. The genetic predisposition to RA is mainly determined by alleles of the main histocompatibility complex class II or HLA from the HLA-DRB1*04 alleles that encode the defective epitope in position 70–74, which is critical for the correct antigen presentation to T cells. RA-associated substitutions in codons 11 and 13 of the same gene have also been described, as well as substitutions in codon 9 of the HLA-B and some other alleles predisposing to RA and autoimmune disorders at the HLA loci. In addition to these polymorphisms, more than 100 non-HLA loci have been characterized as associated with RA. These are mainly polymorphisms in the cytokines (chemokines) and their receptors, costimulatory molecules, and components of intracellular signaling pathways that regulate proliferation and differentiation of lymphocytes (*IL1*, *IL10*, *IL2RA*, *CCR6*, *TNFAIP3*, *STAT4*, *PTPN22*, *PADI4*, *AIRE,* and others) [2].

Genetic predisposition determines not only the risk of RA development and the severity of its clinical manifestations, but also the effectiveness of DMARD (disease-modifying antirheumatic drugs) therapy. The most recommended among them is the conventional synthetic DMARD (csDMARD) methotrexate, which is prescribed after the RA diagnosis as the first line therapy. For patients with no clinical remission after 3–6 months, the second line therapy is administered. It could be a combination of methotrexate with another csDMARD or substitution of methotrexate by biological DMARD (bDMARDs) if a patient has unfavorable prognosis with a high titer of anti-citrulline antibodies, rheumatoid factor, and severe joint damage. The bDMARDs include inhibitors of tumor necrosis factor (TNF), interleukin 6 (IL6), and costimulatory molecules. In case of further RA progression, the third-line therapy is administered. It is represented by the switch to another bDMARD or a combination therapy [3]. Currently, polymorphisms are described in the genes of xenobiotic metabolism, DMARD target molecules (ligands and receptors) and components of signaling pathways associated with the efficacy and toxicity of methotrexate (*MTHFR*, *ABCB1*, *ATIC*, *MTR*, *FPGS*, *SLC19A1*, *TYMS*, *AMPD1*), TNF inhibitors (*TNFA*, *TNFRSF1B*, *TNFR1/2*, *TLR1/5*, *FCGR2A*, *PTPRC*, *NUBPL*), and IL6 inhibitors (*IL6R*, *IL6*) [2,4,5,6].

In early 2014, the results of applied research of a targeted IL6 inhibitor olokizumab were published. Olokizumab is a humanized monoclonal antibody, which is specific to IL6. It binds to the IL6 site that interacts with the CD130 co-receptor and thus blocks the hexameric complex of IL6, IL6R, and CD130 assembly, which is necessary for signal transmission into the cell [7]. Olokizumab successfully passed through phase I of a randomized clinical trial, where the pharmacogenetic, pharmacokinetic, and immunogenic properties of the drug were evaluated in healthy volunteers [8]. Further, its dosage was investigated in RA patients with a similar study of tocilizumab taken as an initial model [9]. Phase II of the study was performed on 119 Asian patients and revealed a significantly more frequent response to olokizumab in comparison with placebo in patients with severe RA who were resistant to TNF inhibitors [10]. The remarkable thing is that the efficacy and safety of olokizumab and sirucumab (antibodies to the IL6 ligand), tocilizumab, and sarilumab (antibodies to the IL6R receptor) were similar at the time of the abovementioned clinical studies. Therefore, searching for genetic predictors could be suggested to personalize the administration of the bDMARDs [11]. Currently, olokizumab is a licensed drug of the R-Pharm group of companies (Russia) that is registered as a drug for RA treatment. Its efficacy was demonstrated in the CREDO2 investigation in RA patients who progressed despite of treatment with methotrexate. Currently, the efficacy of olokizumab and TNF inhibitors (adalimumab) in methotrexate-resistant RA patients is comparable to CREDO3/4 [12]. The aim of our study was to compare the olokizumab efficacy in methotrexate-resistant Russian patients, depending on the polymorphisms of the *IL6*, *IL6R*, *HLA-DRB1/B*, *IL17A*, *IL23R*, *TNFAIP3*, and *TNFA*, and 50 other RA candidate genes, to identify potential allelic variants with prognostic significance.

## 2. Results

### 2.1. Characteristics of Olokizumab Efficacy and Safety

The average alteration in DAS28-CRP in the patients was −2.48 (up to 1.096 points on average) by week 12, and −2.85 (up to 1.042 points on average) by week 24. The response according to DAS28-CRP (<3.2 points on the scale) was achieved in 54/125 (43.2%) RA patients by week 12, and in 67/125 (53.6%) patients by week 24. American College of Rheumatology 20% improvement response criteria (ACR20) was achieved in 89/125 (71.2%) RA patients by week 12, and in 104/125 (83.2%) patients by week 24. According to the same criteria, a 50% improvement in clinical condition (ACR50) was achieved in 57/125 (45.6%) patients by week 12 and in 67/125 (53.6%) patients by week 24, respectively. The average change (with standard deviation) of the RA severity according to the HAQ-DI scale in comparison with the baseline level was −0.5588 (0.4958) by week 12, and −0.6331 (0.5647) by week 24. Non-responders were determined as subjects in any treatment group who did not improve by at least 20% in both swollen and tender joint counts (66–68 joints) by week 14.

At least one adverse event related to infectious complications was registered in 19/125 (15.2%) patients. Manifestations of hepatotoxicity were detected in 10/125 (8.0%) participants; cases of increased activity of alanine aminotransferase (ALT) or aspartate aminotransferase (AST) exceeding the upper limit by more than 1.5 times were reported in 48/125 (38.4%) of the studied patients.

### 2.2. Associations between Polymorphisms and Characteristics of Olokizumab Efficacy and Safety

The response to olokizumab therapy in comparison to the reference category was estimated according to the clinical scales by calculating the odds ratio (OR) and the *p*-value for testing differences between genotypes, estimated by using logistic regression for an unadjusted and adjusted sample. The adjusted model included the following factors: gender, age, body weight (kg), duration of the disease (years), and baseline disease activity (DAS28-CRP). The models of inheritance are considered: dominant, recessive, codominant, over-dominant, and log-additive. If there were genotype associations of several inheritance models and sample correction, the models with the highest OR for the positively associated genotype or, conversely, the lowest for the negatively associated genotype, preferably for the dominant or recessive type of inheritance in the adjusted sample, were selected. An analysis of the early response to olokizumab at week 12 demonstrated the following results with an improvement in the clinical course according to the ACR20 and ACR50, respectively, as well as DAS28-CRP (Table 1).

A number of polymorphisms have demonstrated similar associations when evaluated according to the different clinical scales. Thus, the T allele of the *FPGS* rs10987742 polymorphism is associated with the response to olokizumab in the dominant model, whereas the T alleles of the *PADI4* rs2240336 and *IL6R* rs2228145 in the dominant model and A/A genotype of the *ABCC1* rs3784864 polymorphism and the *AMPD1* rs17602729 polymorphism in the recessive model are associated with olokizumab resistance.

The ANOVA analysis revealed a predictive value of the polymorphic variants *FPGS* rs10987742 and *PADI4* rs2240336 (*p* = 0.02183) when analyzing three polymorphisms significantly associated with DAS28-CRP response at week 12. Their combination was further analyzed by logistic regression and ROC analysis. It was demonstrated that genotyping rs10987742 and rs2240336 gives an area under curve (AUC) of 0.6435 (*p* = 0.006). The inclusion of the *IL6R* rs2228145 polymorphism, as well as the alleles HLA-B*27 and HLA-DRB1*04, associated with a risk of RA development in the studied population [13,14], allows to increase the AUC to 0.6831 (*p* = 0.0009).

However, the main calculations were performed using the results at week 24 of olokizumab therapy, when the systematic long-term effect of the drug could be assessed. When analyzing the late response to olokizumab at week 24, the following results were obtained with an improvement in the clinical course according to the scores of ACR20 and ACR50, respectively, as well as DAS28-CRP (Table 2). The *PADI4* SNPs that were linked with an allele having a larger OR were excluded. Analysis of the linkage disequilibrium here and further was performed by using LDlink software v.5.2 (https://ldlink.nci.nih.gov/, accessed on 11 March 2022), D > 0.95. The SNPs rs2301888, rs2240335, and rs2240336 were excluded from Table 2.

**Figure 1:**
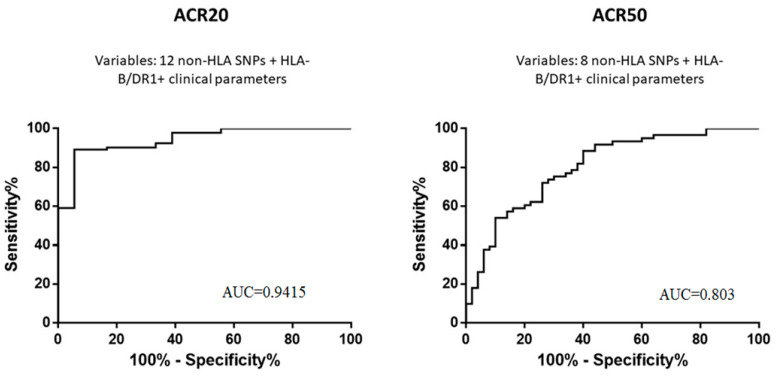
Predictive value of the combinations of polymorphisms and clinical factors in the response to RA therapy with olokizumab.

The analysis of polymorphism combinations that are associated with ACR20 by using logistic regression and ROC analysis revealed an AUC 0.8867 (95% CI: 0.8205–0.9529; *p* < 0.0001). When the alleles HLA-B*27 and HLA-DRB1*04 were additionally included in the model, the AUC increased to 0.9059 (95% CI: 0.8362–0.9756; *p* < 0.0001). The maximal predictive value of the ACR20 response to olokizumab at week 24 was achieved by the inclusion of clinical factors (age, duration of the disease at the time of inclusion in the study, and initial RA activity according to the DAS28-CRP) with genetic polymorphisms: AUC = 0.9415 (95% CI: 0.8902–0.9927; *p* < 0.0001) (Figure 1).

Based on the multivariate ANOVA results, polymorphisms were selected to build a classification tree according to the ACR20 score, namely *TNFRSF1A* rs1974226, *IL23R* rs7539625, and *CCR6* rs3093024. The OR score was 18 (95% CI: 5.201–62.29), with sensitivity of 95.19% and specificity of 47.62% (*p* < 0.0001); the prognostic value of a positive result was 87.2%. In the analysis of polymorphism combinations associated with ACR50 using logistic regression and ROC analysis, an AUC was 0.7696 (95% CI: 0.6832–0.8559; *p* < 0.0001). The inclusion of the alleles HLA-B*27 and HLA-DRB1*04 in the model increased the AUC to 0.7997 (95% CI: 0.7172–0.8821; *p* < 0.0001). The maximal predictive value of the ACR50 response to olokizumab by the end of week 24 was achieved when clinical factors were also included in the model, as for ACR20 (Figure 1). In this case, the AUC for the ACR50 was 0.803 (95% CI: 0.7209–0.8850; *p* < 0.0001). ANOVA analysis for the ACR50 was performed using the same method and demonstrated the advisability of the polymorphisms *ABCC1* rs3784864, *IL1RN* rs419598, *IL18* rs360722, *DHODH* rs3213422, and *PADI4* rs2301888 inclusion in the calculation. The OR score was 5.59 (95% CI: 2.582–12.10) with a sensitivity of 74.63% and specificity of 65.52% (*p* < 0.0001); the prognostic value of a positive result was 70.4%.

ANOVA analysis revealed the predictive value of a combination of polymorphic variants *PADI4* rs1748032 and *IL2RB* rs3218253 (*p* = 0.02183) when analyzing polymorphisms with a DAS28-CRP response at week 24. When the polymorphisms *IL1B* rs16944, *TNFRSF1A* rs1800692, and *IL1RN* rs419598, and alleles HLA-B*27 and HLA-DRB1*04 were additionally included, the result of logistic regression and ROC analysis for the combinations of polymorphisms was an AUC of 0.6894 (*p* = 0.0005). The increase in AUC was achieved by the inclusion of clinical parameters identical to those considered in the two previous models in the calculation: AUC = 0.7752 (95% CI: 0.6874–0.8630; *p* < 0.0001). The OR score in this model was 6.63 (*p* = 0.0025), and the predictive value of a positive result was 72.07%.

The presented data confirm that the model, including all the polymorphisms associated with the response to olokizumab (together with the key HLA-B/DRB1 alleles involved in the RA pathogenesis) and the clinical parameters, has a higher prognostic value. Moreover, the most pronounced association among the scores of ACR20/50 and DAS28-CRP at week 24 was observed when calculating according to ACR20, so with a lower threshold level of the drug response.

### 2.3. Associations of Polymorphisms with Safety Indicators of Olokizumab Therapy

The associations of polymorphisms with the risk of adverse events during olokizumab therapy in RA patients were also evaluated (Table 3). The safety of olokizumab was assessed by the appearance of at least one adverse event from the group “Infections” or “Potential hepatotoxicity” during the olokizumab therapy. Potential hepatotoxicity was estimated according to the protocol of clinical trial CL04041022 as events corresponding to any of the following criteria: ALT > 3 × ULN (upper limit of normal) and total bilirubin > 2 × ULN; ALT > 8 × ULN at any time, regardless of the level of total bilirubin or concomitant symptoms; ALT > 5 × ULN for ≥2 weeks, regardless of the level of total bilirubin or concomitant symptoms; and ALT > 3 × ULN with concomitant symptoms of liver pathology (fatigue, nausea, vomiting, pain or soreness in the upper right quadrant, or rash).

It should be noted that the same genotypes are often associated with the olokizumab efficiency and severity of side effects, especially hepatotoxicity. Thus, 6/13 alleles (genotypes) that are associated with the response to olokizumab by ACR20 at week 24 in different combinations demonstrated an association with adverse events. According to the linkage disequilibrium analysis, the *PADI4* rs2240340, rs11203366, rs230188, rs11203367, and rs2240335 were excluded. The results suggest the feasibility of a separate assessment of the efficacy and probability of adverse events of olokizumab therapy when using genotyping and calculating the relative risk in the Russian population.

## 3. Discussion

This study demonstrated associations of olokizumab efficacy in RA therapy with polymorphic variants of 19 genes. Among them are genes encoding interleukins and their receptors (*IL6R*, *IL1B*, *IL23R*, *IL17A*, *IL18*, and *IL1RN*), components of the TNF signaling pathway and chemokine receptors (*TNFRSF1A*, *TNFAIP3*, *CCR6*, *TLR5*), molecules of the main histocompatibility complex (HLA-B and HLA-DRB1), and proteins involved in the detoxification of xenobiotics (for example, *ABCB1* and *ABCC1*). The analysis of polymorphic variant combinations revealed the maximal predictive value according to the ACR20 at week 24 of olokizumab therapy for the combination of alleles HLA-DRB1*04 and HLA-B*27 together with alleles (genotypes) of non-HLA genes and such clinical parameters such as the age of a patient, RA severity, and duration at the time of inclusion in the study. Among non-HLA genes, the largest number of olokizumab-associated SNPs (4/11) was localized in *PADI4*. However, one of them was excluded after the linkage disequilibrium analysis. *PADI4* is considered as one of the most prominent RA candidate genes. It encodes peptidyl–arginine–deaminase that converts arginine amino acid residues to citrulline, and deregulation of this process can contribute to the formation of antibodies to citrulline. Increased PADI4 activity was observed in the inflammatory infiltrate, where it leads to abnormal citrullinization of fibrin filaments [15]. Among the three polymorphisms that independently demonstrated associations with the investigated clinical features are rs1748032 and rs2240336. They are intronic variants that could be linked to functionally significant alleles of the other *PADI4* SNPs included in the study with less pronounced OR, or other alleles of this or closely localized genes. The rs874881 polymorphism is a p.G 112A missense variant, which does not have an unambiguous pathogenicity value in ClinVar (https://www.ncbi.nlm.nih.gov/clinvar/, accessed on 11 March 2022). Nevertheless, the association of rs874881 with RA in some populations and its functional significance was demonstrated in several studies [16,17,18].

Another RA candidate gene *TNFAIP3* was characterized by the rs6920220 allele A polymorphism, which is negatively associated with the response to olokizumab in the dominant model. *TNFAIP3* is localized in the 6q23 region and encodes the ubiquitin-modifying enzyme A20 that is a component of the NF-kB signaling pathway [19]. The rs1143634 *IL1B* and rs1974226 *IL17A* demonstrated negative associations with the olokizumab efficacy in the recessive model, and these genes were also previously described as RA candidate genes [20,21]. The rs7539625 *IL23R* A allele was associated with the response to olokizumab and a significant increase in OR in the dominant model.

It is interesting to note that the rs2228145 *IL6R* that is the gene encoding the olokizumab target was associated with an early response to the drug at week 12 according to the scores ACR20 and DAS28-CRP, but not with a late response at week 24. Probably, the variants in IL6R structure play a role in direct interaction with the IL6 ligand and olokizumab, thus affecting its efficacy at the beginning of treatment, whereas the prolonged use of the drug and its systemic effect and genetically determined differences in the activity of other cytokines and their receptors already result in predominance in the intensity of clinical manifestations. Further study of interactions of different IL6R variants with ligands and coreceptors is important not only for the olokizumab efficacy study, but also for its potential improved analogues in the future, considering current capabilities of new drug computer design. For example, an in silico investigation of 889 mutant variants of the olokizumab light chain revealed eight antibody variants that could more effectively block IL6 interaction with receptors [22]. Besides, the study NCT02760368 demonstrated the effect of the dose and frequency of olokizumab administration on the therapy efficacy [23]. However, because of the limited sample size of 125 patients in our study, associations of the polymorphisms with the frequency of olokizumab administration were not analyzed. The study was not aimed at comparing the biochemical parameters between responders and non-responders for the same reason.

When elaborating a panel of prognostic genetic markers of olokizumab efficacy, it is appropriate to consider an epigenetic mechanism contribution. Thus, our earlier analysis of microRNA (miRNA) expression in 103 patients from the studied sample revealed associations of miR-26b, miR-29, miR-451, and miR-522 with olokizumab efficacy in RA patients [24]. Administration of bDMARD, including olokizumab, considering personal biochemical and genetic characteristics of a patient, could help to optimize the cost of treatment for an RA patient [25]. A comprehensive assessment of HLA and non-HLA genes, polymorphic variants and population characteristics, and miRNA expression, together with clinical parameters of RA intensity at the beginning of olokizumab therapy, may allow to elaborate a panel of RA prognostic markers for Russian patients.

Although the studied sample is small and includes 125 patients, these patients were thoroughly examined according to clinical and laboratory parameters and represent one of the most numerous Slavic RA patient samples. The sample was already investigated in the study of genetic factors of predisposition to the development of the disease and the severity of its clinical manifestations [21]. In the future, it is possible to expand the sample by continuing genetic research within the framework of the ongoing project. Besides, an additional study of the mRNA expression of genes whose SNPs have been shown to be associated with the olokizumab efficiency may help to better characterize the functional role of these associated loci in the mechanism of the drug response.

It should also be noted that this is the first population-oriented study of presumable genetic efficacy predictors of olokizumab, in contrast to methotrexate, for example, where dozens of similar works and meta-analyses have been published [2,4]. In this regard, it may be of interest as a starting point for further broader studies of polymorphisms associated with the response to olokizumab in RA patients of Eastern European populations.

## 4. Materials and Methods

### 4.1. Sample Characteristics

Samples from 125 Russian patients with RA, progressing during the methotrexate therapy, were studied (109 women and 16 men). Adults were eligible for inclusion if they had active RA (swollen joint count ≥ 6 (66-joint count), tender joint count ≥ 6 (68-joint count) and CRP > 6 mg/L) classified by the American College of Rheumatology/European League Against Rheumatism 2010 revised classification criteria for at least 12 weeks prior to screening and had an inadequate response to treatment with methotrexate for at least 12 weeks at a dose of 15–25 mg/week (or ≥10 mg/week if intolerant to higher doses). The dose and route of administration of methotrexate must have been stable for at least 6 weeks. In the clinical trial CL04041022, patients with moderate to severe RA insufficiently controlled by methotrexate therapy were treated with olokizumab (subcutaneous solution 160 mg/mL; R-Pharm, Moscow, Russia) at a dose of 64 mg once per 4 weeks or 64 mg once per 2 weeks over a 24-week period. During the treatment period, the patients continued background therapy with methotrexate in a stable mode at a dose of 15–25 mg per week (or ≥10 mg per week, in the case of documented intolerance to higher doses) without changing the method of the drug administration. All the patients required concomitant therapy with folic acid at a dose ≥ 5 mg/week or its equivalent. The parameters of the analyzed sample at the time of inclusion of patients in the study are summarized in Table 4.

In the study cohort, 59 patients (47.2%) were treated with olokizumab every 2 weeks, and 66 patients (52.8%) were treated every 4 weeks. The majority of patients (124/125, 99.2%) completed treatment with olokizumab in accordance with the protocol. One patient (CL04041022-0131-0008) completed therapy earlier on day 58 due to the development of an undesirable phenomenon (increased alanine aminotransferase (ALT) and aspartate aminotransferase (AST)) and was excluded from the study on day 239. According to the protocol of the clinical trial, the patient was allowed to prescribe other antirheumatic drugs (sulfasalazine and/or hydroxychloroquine) as a supplement to the olokizumab, if there was no response to therapy by week 14 (no positive dynamics in more than 20% of painful and swollen joints). Twelve (9.6%) patients required the prescription of additional DMARDs after week 14.

### 4.2. DNA Extraction

Two to three milliliters of blood from each patient was collected into K3EDTA tubes. Genomic DNA was extracted from blood using a DNA-sorb-B kit (CRIE, Moscow, Russia).

### 4.3. HLA Genotyping

Genotypes of the HLA-DRB1 locus were determined by real-time polymerase chain reaction (RT-PCR) using the HLA-DNA-Tech kit (DNA Technology, Moscow, Russia), which allows differentiating groups of DRB1 alleles 01, 03, 04, 07, 08, 09, 10, 11, 12, 13, 14, 15, and 16. The carriers of the B27 allele in the HLA-B locus were detected with the HLA-B27 kit of the same manufacturer. RT-PCR was performed on the DT-Prime thermal cycler (DNA technology, Russia). The software provided with the kits was used for the result visualization.

### 4.4. Non-HLA Genotyping

Sequencing of polymorphic variants localized outside the sites of major mutations in HLA genes was performed using the AmpliSeq panel IAD177464_185 (Thermo Fisher Scientific, Waltham, MA, USA). This multigenic panel includes coding sequences of *IL6*, *IL6R*, *TNFRSF1A*, *CTLA4*, *IL10*, *IL23R*, and *PADI4*, partially *HLA-DRB1* and *HLA-B* genes, as well as 60 short genome fragments with polymorphic variants (predominantly in the coding regions of the genes), which, according to the literature, are associated with RA development and efficacy of treatment. A complete list of analyzed sequences is available on request (IAD177464_185_coverage_summary.csv). The libraries were prepared using the Ion AmpliSeq Library Kit 2.0. The chips 540 were prepared at the IonChef station, followed by semiconductor sequencing on the IonS5 Systems device (Thermo Fisher Scientific, Waltham, MA, USA). The MAFs of the non-HLA SNPs that demonstrated significant associations with the efficacy and safety of olokizumab are presented in the supplementary files (Supplementary Table S1).

### 4.5. Statistical Data Analysis

The mean changes and standard deviations were determined according to the clinical scales ACR20, ACR50, and DAS28-CRP. The response to olokizumab in RA patients was evaluated by using logistic regression, ROC analysis, and multivariate ANOVA analysis separately for polymorphisms that were identified by using NGS panel and did not include HLA, following the inclusion of the HLA-B*27 and HLA-DRB1*04 alleles, as well as with the incorporation into the model of the parameters of age, duration of the disease, and its intensity at the time of inclusion in the study.

## 5. Conclusions

Thus, in this study of a sample of Russian patients with RA, resistant to methotrexate and treated with olokizumab, associations of polymorphic variants of 19 genes with olokizumab efficacy based on the criteria ACR20, ACR50, and DAS28-CRP were revealed, as well as associations of 11 loci with adverse events during the treatment. However, these data are characterized by a weak association, presumably because of the small volume of the non-responders subgroup. Data analysis using logistic regression, ROC curves, and multivariate ANOVA demonstrated that the combination of alleles *04 of the HLA-DRB1 and *27 of the HLA-B with alleles (genotypes) of 11 non-HLA genes and clinical parameters (patient age, intensity, and duration of RA at the time of inclusion in the study) has more prominent association with response to olokizumab, if using ACR20 at week 24 as the response criterion. For a deeper understanding of molecular genetic factors affecting the olokizumab efficacy, a further investigation should be performed on HLA and non-HLA polymorphic variants and other factors of the RA molecular pathogenesis, pharmacodynamics, and pharmacokinetics of olokizumab, estimating the disease severity according to the clinical evaluation scores and the population characteristics of patients.

## Figures and Tables

**Table 1:** Associations of polymorphic variants with the efficacy of olokizumab therapy at week 12.

Gene	SNP	Genotype	OR (CI 95%)	*p*	Model	Sample	Effect
ACR20							
*TNFRSF1A*	rs767455	C/C	5.62 (1.42–22.29)	0.0140	R	A	↑
*IL23R*	rs1884444	T/T	5.47 (1.65–18.14)	0.0055	R	A	↑
*IL1B*	rs1143634	G/A	2.90 (1.05–8.01)	0.0403	OD	A	↑
*ABCB1*	rs2032582	A/C-C/C	2.63 (1.00–6.91)	0.0493	D	UA	↑
*FPGS*	rs10987742 *	C/T	2.47 (1.07–5.68)	0.0334	OD	UA	↑
*AMPD1*	rs17602729 *	A/A	0.12 (0.02–0.89)	0.0384	R	A	↓
*PADI4*	rs2240336 *	C/T-T/T	0.28 (0.10–0.78)	0.0142	D	A	↓
*ABCC1*	rs3784864 *	A/A	0.31 (0.13–0.75)	0.0095	R	A	↓
*IL6R*	rs2228145 *	A/C-C/C	0.38 (0.16–0.94)	0.0372	D	A	↓
*TLR5*	rs5744174	A/G	0.41 (0.17–0.98)	0.0458	OD	A	↓
ACR50							
*IL17A*	rs1974226	C/T-T/T	2.38 (1.08–5.26)	0.0319	D	UA	↑
*IL1B*	rs16944	G/G	2.18 (1.02–4.66)	0.0447	R	A	↑
*ABCB1*	rs1045642	A/G	2.18 (1.06–4.49)	0.0349	OD	UA	↑
*AMPD1*	rs17602729 *	G/A-A/A	0.38 (0.16–0.91)	0.0309	D	A	↓
*ABCC1*	rs3784864 *	A/A	0.43 (0.18–1.00)	0.0488	R	UA	↓
*IL1B*	rs1143623	C/G-G/G	0.46 (0.22–0.99)	0.0457	D	A	↓
DAS28-CRP							
*FPGS*	rs10987742 *	C/T-T/T	2.20 (1.05–4.61)	0.0359	D	UA	↑
*IL6R*	rs2228145 *	A/C-C/C	0.38 (0.17–0.84)	0.0170	D	A	↓
*PADI4*	rs2240336 *	C/T-T/T	0.39 (0.17–0.89)	0.0247	D	A	↓

OR—odds ratio, CI—confidence interval, CRP—C-reactive protein, SNP—single nucleotide polymorphism, p—probability that the null hypothesis is correct, *—similar results were obtained according to different clinical scales; inheritance models: D—dominant, R—recessive, OD—over-dominant; sample: UA—unadjusted, A—adjusted; arrows indicate association with high and low olokizumab efficacy.

**Table 2:** Associations of polymorphic variants with the efficacy of olokizumab treatment at week 24.

Gene	SNP	Genotype	OR (95% CI)	*p*	Model	Sample	Effect
ACR20							
*PADI4*	rs1748032 *	T/T	5.23 (1.09–25.04)	0.0382	CD	A	↑
*IL23R*	rs7539625	G/A-A/A	3.07 (1.03–9.15)	0.0446	D	A	↑
*PADI4*	rs2240336	C/T-T/T	3.00 (1.07–8.47)	0.0375	D	A	↑
*PADI4*	rs2301888 *	G/A-A/A	2.94 (1.06–8.15)	0.0387	D	A	↑
*PADI4*	rs2240335	C/A-A/A	2.85 (1.04–7.78)	0.0415	D	A	↑
*IL17A*	rs1974226	A/A	0.12 (0.02–0.79)	0.0274	R	A	↓
*IL1B*	rs1143634 *	A/A	0.21 (0.05–0.93)	0.0400	R	A	↓
*GLCCI1*	rs37972	C/C	0.23 (0.08–0.64)	0.0047	R	A	↓
*DHODH*	rs3213422 *	A/C	0.30 (0.11–0.88)	0.0283	OD	A	↓
*CCR6*	rs3093024	A/G	0.32 (0.12–0.87)	0.0261	OD	UA	↓
*TNFAIP3*	rs6920220	G/A-A/A	0.35 (0.13–0.95)	0.0393	D	A	↓
ACR50							
*IL18*	rs360722	G/G	2.75 (1.09–6.94)	0.0322	R	A	↑
*IL1RN*	rs419598 *	T/C	2.24 (1.01–5.01)	0.0483	OD	A	↑
*ABCB1*	rs2032582	A/C	2.18 (1.03–4.63)	0.0418	OD	A	↑
*PADI4*	rs2301888 *	G/A	2.17 (1.00–4.70)	0.0489	OD	A	↑
*IL1B*	rs1143634 *	A/A	0.13 (0.02–0.83)	0.0313	R	A	↓
*TLR5*	rs5744174	A/G-G/G	0.31 (0.12–0.77)	0.0124	D	A	↓
*DHODH*	rs3213422 *	A/C	0.41 (0.19–0.88	0.0218	OD	A	↓
*ABCC1*	rs3784864	A/A	0.42 (0.18–0.97)	0.0428	R	A	↓
DAS28-CRP							
*PADI4*	rs1748032 *	C/T-T/T	4.78(1.30–17.54)	0.0184	D	A	↑
*IL1B*	rs16944	G/G	2.43 (1.10–5.35)	0.0279	R	A	↑
*IL1RN*	rs419598 *	C/C	0.21 (0.04–0.94)	0.0412	R	A	↓
*TNFRSF1A*	rs1800692	A/G-G/G	0.37 (0.14–1.00)	0.0495	D	A	↓

OR—odds ratio, CI—confidence interval, CRP—C-reactive protein, SNP—single nucleotide polymorphism, p—probability that the null hypothesis is correct, *—similar results were obtained according to different clinical scales; inheritance models: D—dominant, CD—codominant; R—recessive, OD—over-dominant; sample: UA—unadjusted, A—adjusted; arrows indicate association with high and low olokizumab efficacy.

**Table 3:** Associations of polymorphic variants with the safety of olokizumab.

Gene	SNP	Genotype	OR (95% CI)	*p*	Model	Risk
Infectious complications						
*IL2RB*	rs3218253	A/A	8.87 (1.39–56.54)	0.0210	R	↑
*DHODH*	rs3213422	C/C	3.53 (1.31–9.54)	0.0128	R	↑
*TNFRSF1A*	rs767455 *	T/C	3.03 (1.09–8.41)	0.0331	OD	↑
*IL17A*	rs1974226 *	C/T-T/T	0.16 (0.03–0.93)	0.0406	D	↓
*IL1B*	rs16944	G/G	0.22 (0.06–0.88)	0.0323	CD	↓
*TNFRSF1A*	rs1800692	G/G	0.26 (0.08–0.87)	0.0293	R	↓
Potential hepatotoxicity						
*IL17A*	rs1974226 *	C/T-T/T	10.44 (2.37–46.03)	0.0019	D	↑
*PADI4*	rs874881	G/C	7.40 (1.25–43.63)	0.0270	OD	↑
*IL2RA*	rs2104286	C/C	7.29 (1.19–44.56)	0.0316	R	↑
*IL18*	rs360718	C/C	5.91 (1.03–33.84)	0.0460	R	↑
*IL23R*	rs1884444	G/T	4.57 (1.05–19.84)	0.0426	OD	↑
*PADI4*	rs2240340	T/C	2.43 (1.16–5.10)	0.0184	OD	↑
*PADI4*	rs11203366	G/A	2.31 (1.10–4.83)	0.0263	OD	↑
*TNFRSF1A*	rs767455 *	T/C	2.10 (1.01–4.37)	0.0467	OD	↑
*STAT4*	rs7574865	T/G-G/G	0.11 (0.02–0.53)	0.0062	D	↓
*PADI4*	rs2240335	A/A	0.14 (0.02–0.82)	0.0291	R	↓
*PADI4*	rs2301888	A/A	0.14 (0.02–0.82)	0.0291	R	↓
*PADI4*	rs2240336	T/T	0.22 (0.05–0.90)	0.0357	R	↓
*AMPD1*	rs17602729	G/A-A/A	0.39 (0.16–0.93)	0.0345	D	↓
*PADI4*	rs11203367	C/C	0.39 (0.16–0.93)	0.0345	R	↓
*IL1B*	rs1143634	G/A-A/A	0.43 (0.20–0.94)	0.0340	D	↓

OR—odds ratio, CI—confidence interval, CRP—C-reactive protein, SNP—single nucleotide polymorphism, p—probability that the null hypothesis is correct, *—similar results were obtained according to different clinical scales; inheritance models: D—dominant, CD—codominant; R—recessive, OD—over-dominant; arrows indicate association with high and low risk of adverse events.

**Table 4:** Clinical and laboratory characteristics of the 125 RA patients.

№	Variables	Statistical Values
	Anthropometric data	
1	Age (mean ± SD; min/max), years	50.4 ± 13.14; 22/82
2	Weight (mean ± SD ± CO; min/max), kg	73.63 ± 16.36; 41/131
	RA severity	
3	Disease duration (mean ± SD; min/max), years	5.91 ± 1.214; 2.61/11.16
4	Disease severity based on DAS28- CRP (mean ± SD; min/max), scores moderate (DAS28-CRP > 3.2 to ≤5.1), subjects high (DAS28-CRP > 5.1), subjects	5.94 ± 0.64; 4.5/8.1 10 (8%) 113 (90.4%)
5	Disease severity based on CDAI (mean ± SD; min/max), scores	39.43 ± 8.7; 24.8/69.3
6	Disease severity based on HAQ-DI (mean ± SD; min/max), scores	1.68 ± 0.5; 0.13/2.86
	Laboratory parameters	
7	RF level (mean ± SD; min/max), IU/mL among them ≥ 15 IU/mL:	192.2 ± 240.95; 7/1540 84% (105/125)
8	Anti-CCP level (mean ± SD; min/max), IU/mL among them > 10 ME/mL	664.35 ± 999.31; 0.4/6044.8 80.8% 101/125
9	CRP level (mean ± SD; min/max), mg/mL	21.0 ± 20.83; 1/120

Anti-CCP—anti-cyclic citrullinated peptide antibodies, RA—rheumatoid arthritis, RF—rheumatoid factor, SD—standard deviation, CRP—C-reactive protein.

## Data Availability

Not applicable.

## Supplementary Materials

The following supporting information can be downloaded at: https://www.mdpi.com/article/10.3390/jpm12040641/s1, Table S1: The MAFs of the non-HLA SNPs that demonstrated significant associations with the efficacy and safety of olokizumab.
