# Supplementary material for: Genetic and Clinical Factors Associated with Olokizumab Treatment in Russian Patients with Rheumatoid Arthritis
Source: J Pers Med. 2022 Apr 15;12(4):641. doi: 10.3390/jpm12040641 (PMC9024465; doi:10.3390/jpm12040641)
Supplement: Supplementary file 1 [file jpm-12-00641-s001.zip › jpm-1686511-supplementary.pdf]

Supplementary Table S1. The MAFs of the non-HLA SNPs that demonstrated significant associations with the efficacy and safety of olokizumab.

Gene	SNP	Genotype	MAF Total GnomAD	MAF European (Non-Finnish)
TNFRSF1A	rs767455	T/C	0.399	-
TNFRSF1A	rs1800692	A/G	0.291	0.227
TNFAIP3	rs6920220	G/A	0.158	0.208
DHODH	rs3213422	A/C	0.446	0.507
FPGS	rs10987742	C/T	0.242	0.231
AMPD1	rs17602729	G/A	0.086	0.129
ABCC1	rs3784864	A/A	0.404	0.517
ABCB1	rs2032582	A/C	0.539	0.549
ABCB1	rs1045642	A/G	0.439	-
TLR5	rs5744174	A/G	0.385	0.437
GLCCI1	rs37972	T/C	0.362	-
CCR6	rs3093024	A/G	0.389	0.294
PADI4	rs874881	G/C	0.478	-
PADI4	rs2240336	C/T	0.428	0.402
PADI4	rs2301888	G/A	0.317	0.347
PADI4	rs2240335	C/A	0.389	0.349
PADI4	rs2240340	T/C	0.455	-
PADI4	rs11203366	G/A	0.449	-
PADI4	rs1748032	C/T	0.632	0.649
PADI4	rs11203367	C/T	0.441	-
IL23R	rs1884444	G/T	0.486	-
IL23R	rs7539625	G/A	0.326	-
IL17A	rs1974226	C/T	0.153	0.18
IL18	rs360722	A/G	0.183	0.173
IL18	rs360718	A/C	0.258	-
IL6R	rs2228145	A/C	0.379	0.396
IL2RA	rs2104286	T/C	0.179	-
IL2RB	rs3218253	G/A	0.218	0.268
IL1RN	rs419598	T/C	0.256	0.273
IL1B	rs1143634	G/A	0.192	0.241
IL1B	rs16944	G/A	0.421	0.332
IL1B	rs1143623	C/G	0.240	-
